# Salivary biomarkers as key to monitor personalized oral healthcare and precision dentistry: A scoping review

**DOI:** 10.3389/froh.2022.1003679

**Published:** 2022-09-22

**Authors:** Pune Nina Paqué, Jenni Hjerppe, Anina N. Zuercher, Ronald E. Jung, Tim Joda

**Affiliations:** ^1^ Clinic of Reconstructive Dentistry, Center of Dental Medicine, University of Zurich, Zurich, Switzerland; ^2^ Department of Reconstructive Dentistry, University Center of Dental Medicine, University of Basel, Basel, Switzerland

**Keywords:** saliva, oral infections, gingivitis, periodontitis, diagnostics

## Abstract

Personalized Oral Healthcare has recently become the new trend word in medicine and dentistry. In this context, saliva diagnostics using various biomarkers seem to be the gateway to personalized dental diagnostics and therapy. But the terminology is not (yet) uniformly defined, furthermore it is unclear to what extent which salivary markers play a relevant role in the therapeutic decision making. In this Scoping Review, an electronic search was conducted in PubMed and Web of Science databases using medical subject headings (MESH terms) “saliva”, “biomarker”, “personality/persons”, and “dentistry”. Only human studies were included, in which repeated salivary measurements were performed to analyze monitoring effects with at least ten patients per group. PRISMA-ScR and Tricco guidelines were followed: (i) to examine what salivary biomarkers have been explored in terms of personalized oral healthcare and precision dentistry, (ii) to investigate the clinical relevance for oral health and its correlation to systemic health, and (iii) to summarize an outlook for future developments based on these results. Out of 899 studies, a total of 57 were included for data extraction in this Scoping Review, mainly focusing on periodontal therapy and patient monitoring. Salivary biomarkers have shown the potential to change the field of dentistry in all dental disciplines as a key for personalized workflows. The increasing interest in dental research is obvious, demonstrated by the growing number of publications in recent years. At this time, however, the predominant discipline is periodontology, which allows biomarker-based monitoring of the disease prevention and progression. The studies included showed heterogeneous methods using manifolds biomarkers. Therefore, no uniformly accepted concept can be presented today. Further clinical research with well-defined outcomes including standardized procedures is necessary.

## Introduction

The maintenance of oral health can be achieved by preventive measures and the early detection and timely treatment of incipient oral infections, namely caries and periodontitis. Both non-communicable diseases are based on a disbalance of microbial biofilms in the oral cavity (1). Preventive measures to combat these pathogenic biofilms on a daily basis were summarized recently in different Consensus Reports (2, 3). Evidently, the most powerful individual tools for prevention and maintenance were based on self-performed oral hygiene and use of cleaning agents containing fluoride. Aside, it seems crucial to control and detect individuals' deviations of the oral health status at an early stage to hinder disease outbreak. While gold standard clinical examinations and radiographs at the dental office may miss developing disorders on a cellular base, other diagnostic tools and modern technical possibilities gain more importance (4, 5). Different biofluids, such as periphery blood, gingival crevicular fluid (GCF), and saliva were evaluated for their diagnostic or prognostic qualification for disease detection (6). The ease and non-invasiveness in saliva collection positions this biofluid as potential alternative to blood testing. Interestingly, the search for saliva diagnostics in different databases demonstrates already a high quantity of clinical investigations using saliva as target vehicle for different biomarkers in oral diseases. Saliva bathes all healthy and diseased oral surfaces in the oral cavity (7, 8). It was shown, that the oral microbiome can be quantified to a certain extent in saliva (9, 10), and even more interesting, host immune response to pathogens can be analyzed nearly in real-time (11). Furthermore, modern technologies allow the detection and quantification of specific substances in low concentrations (4, 12). Today, improvements and further developments in point-of-care devices evolve from technical progress in the era of Covid 19 (13, 14). With that much data and even more technical possibilities, the questions arise, what to actually search for.

Single salivary measurements usually present a condition at a specific timepoint. These measurements can be used to define special patient groups or signature profiles of different diseases and to detect outlier (10). In contrast, repeated salivary measurements provide information on trends, progression of disease, and on response to different treatment strategies (11, 15). Furthermore, it allows the monitoring of patients during maintenance and offers thereby the possibility to retreat timely—if needed. The outcome might help to detect personalized salivary pattern in reaction to different trigger and in future to compute custom-fit therapies for precision dentistry. This might also pave the way for real-time measurements outside the dental office and its implementation in daily life.

Based on these thoughts, the high number of clinical trials using saliva, and on the lack of structure in saliva literature, the three objectives of this Scoping Review are:

• What salivary biomarkers have been investigated in clinical trials in terms of personalized oral healthcare and precision dentistry mapped for disciplines and/or indications (key elements: choice of biomarkers, number of participants, dental disciplines)?
• What has been the clinical relevance for oral health and its correlation to systemic health?
• What can be summarized for a future outlook?

## Methods

The conduct of this review follows the Arksey and O'Malley framework, modified by Levac (16, 17), omitting the consultation step. Reporting follows the PRISMA-ScR statement (18), shown in Supplementary_Data 1; in addition, the preliminary review protocol including PCC-question is available as Supplementary_Data 2.

Dentistry-related clinical trials, written in English, which investigated salivary analysis at different timepoints, and participants at least 18 years, were included. Different timepoints were defined as a minimum of two separate repeated salivary measurements at different periods. Clinical trials investigating plain general medicine subjects, without reference to the oral health status, were excluded. Clinical trials with less than 10 participants per group, and animal studies, as well as review articles were excluded.

An electronic search was performed in the database PubMed and Web of Science for relevant papers using “saliva” as the source of analysis, “biomarker” as concept, and “(personalized) dentistry” or “precision dentistry” as context. Search combinations in PubMed included: ("saliva"[MeSH Terms] OR "saliva"[All Fields] OR "salivas"[All Fields] OR "saliva s"[All Fields] OR "salivary"[All Fields]) AND ("biomarker s"[All Fields] OR "biomarkers"[MeSH Terms] OR "biomarkers"[All Fields] OR "biomarker"[All Fields]) AND ((("person s"[All Fields] OR "personable"[All Fields] OR "personableness"[All Fields] OR "personal"[All Fields] OR "personalisation"[All Fields] OR "personalise"[All Fields] OR "personalised"[All Fields] OR "personalising"[All Fields] OR "personality"[MeSH Terms] OR "personality"[All Fields] OR "personalities"[All Fields] OR "personality s"[All Fields] OR "personalization"[All Fields] OR "personalize"[All Fields] OR "personalized"[All Fields] OR "personalizes"[All Fields] OR "personalizing"[All Fields] OR "personally"[All Fields] OR "personals"[All Fields] OR "persons"[MeSH Terms] OR "persons"[All Fields] OR "person"[All Fields]) AND ("dentistry"[MeSH Terms] OR "dentistry"[All Fields] OR "dentistry s"[All Fields])) OR (("precise"[All Fields] OR "precised"[All Fields] OR "precisely"[All Fields] OR "preciseness"[All Fields] OR "precises"[All Fields] OR "precision"[All Fields] OR "precisions"[All Fields]) AND ("dentistry"[MeSH Terms] OR "dentistry"[All Fields] OR "dentistry s"[All Fields]))). All publications were included until the 30th of June 2022. The search in Web of Science was conducted with the terms “(ALL = (saliva*biomarker* ((person* and dentistry*) or (precis* and dentistry*)))).

Title and abstract screening were performed by three authors (PNP, JH, AZ) using a data chart, which was discussed during a first exploratory screening (Supplementary_Data 3). The following data items were extracted during the full-text search: first author, year of publication, country of origin (= where the study was conducted), dental discipline (e.g., periodontology, implantology, prosthodontics), topic (research question), applied salivary biomarker, monitoring intervals, total number of patients, outcome metrics/conclusion of the study, study design (Table 1). All included studies were categorized to dental disciplines, including a total number of patients per group. Major biomarkers of included studies were specified in frequency of occurrence and in their combination.

**Table 1:** Included studies with extracted data items.

	Study	Year	Country	Dental Discipline	Biomarker	Monitoring intervals	Patients	Study design
1	Al-Hamoudi N (19)	2018	Saudi Arabia	Periodontology	Resistin, IL-6	Baseline, 6 months	137	Observational study
2	Alajbeg IZ (20)	2020	Croatia	TMD	OS	Baseline, 3 and 6 months	34	RTC
3	AlJasser R (21)	2021	Saudi Arabia	Implantology	IL-1β, IL-6, MMP-8, TNF-α, TIMP-1	Baseline, 1, 6 and 12 months	60	Case-control study
4	Aspiras MB (22)	2013	USA	General Dentistry	IL-1β, IL-8, MCP-1, MMP-1, MMP-3, MMP-8, MMP-9, TIMP-1, TIMP-2, TIMP-3, TIMP-4, IL-1ra, NGAL	Baseline, 21 and 49 days	175	RCT (single-blinded)
5	Äyräväinen L (23)	2018	Finland	Periodontology	IL-6, MMP-8, TIMP-1	Baseline, 12 months	124	Observational study
6	Bertl K (24)	2013	Austria	Periodontology	Melatonin	Baseline, 3 months	60	Observational study
7	Bikker FJ (25)	2019	Denmark	Periodontology	Protease activity	-14 d, baseline, 7, 14 and 21 days	42	Observational study
8	Buczko *P* (26)	2017	Poland	Orthodontics	Thio-Barbituric Acid Reacting Substances (TBARS), TOS, SOD, CAT, UA, Peroxidase activity, TAS	Baseline, 1 and 24 weeks	60	Observational study
9	Buduneli N (27)	2006	Turkey	Periodontology	Glutathiode, Ascorbic Acid, TAC, Cotinine	Baseline, 1 month	20	Observational study
10	Chang CH (28)	2018	Taiwan	Periodontology	Oxidative biomarkers, Cu/Zn SOD, MnSOD, TRX1, PRX2	Baseline, 3 months	167	Observational study
11	Cutando A (29)	2013	Spain	General Dentistry	Alkaline Phosphatase, Acid Phosphatase, Osteocalcin, Osteopontin	Baseline, 20 days	60	Observational study
12	Dede FÖ (30)	2013	Turkey	Periodontology	8-Hydroxy-Deoxy-Guanosine (8-OHdG)	Baseline, 10 days, 1 and 3 months	48	Observational study
13	Fine DH (31)	2014	USA	Periodontology	Macrophage Inflammatory Protein (MIP) 1a / 1 b, IL-a, IL-1β, IL-8	Baseline, 6, 12, 18, 24, 30, 36 months	100	Observational study
14	Fujimori K (32)	2021	Japan	Periodontology	miRNAs	Baseline, 2 years	120	Observational study
15	Ghallab N (33)	2010	Egypt	Periodontology	sCD44	Baseline, 1 month	44	Pilot study
16	Gutiérrez-Corrales A (34)	2017	Spain	Oral Surgery	Total protein, IgA, Alpha-Amylase	Baseline, immediately after extraction, 2 h, 7 days	15	Observational study
17	Hassan SH (35)	2015	Egypt	Periodontology	Osteoprotegerin (OPG)	Baseline, 3 and 6 months	30	Observational study
18	Hendek MK (36)	2015	Turkey	Periodontology	8-Hydroxy-Deoxy-Guanosine (8-OHdG), 4-Hydroxy-Nonenal (HNE), enzyme activity of Glutathione Peroxidase (GSH-Px)	Baseline, 1 and 3 months	93	Observational study
19	Hodosy H (37)	2005	Slovak republic	General Dentistry	Thio-Barbituric Acid Reacting Substances (TBARS)	Morning, afternoon, evening, 2 consecutive days	10	Observational study
20	Jentsch H (38)	2004	Germany	Periodontology	Lactoferrin, Lysozyme, Peroxidase activities	Baseline, 14 days	25	Observational study
21	Jenzsch A (39)	2009	Germany	Periodontology	Periopathogens, IL-1β, IL-6, Granulocyte Elastase activity, anti-oxidative and oxidative variables	Baseline, 2, 3 and 6 weeks, 12 months	20	Observational study
22	Justino AB (40)	2017	Brazil	General Dentistry	Salivary total protein, Nitrite, total anti-oxidant capacity, Alpha-Amylase	Baseline, after toothbrushing	14	Observational study
23	Kamodyová N (41)	2013	Slovakia	General Dentistry	Advanced oxidation protein products (AOPP), Thio-Barbituric Acid Reactive Substances (TBARS), Advanced Glycation End Products (AGEs), Ferric Reducing Antioxidant Power (FRAP), total antioxidant capacity	Morning, afternoon, evening, each, before and after toothbrushing	19	Observational study
24	Kibayashi M (42)	2007	Japan	Periodontology	Periopathogens, Prostaglandin E2, Lactoferrin, Albumin, Aspartate Aminotransferase, Lactate Dehydrogenase, Alkaline Phosphatase	Baseline, 4 years	256	Observational study
25	Kim HN (43)	2022	Korea	Periodontology	MMP-3, MMP-8, MMP-9	Baseline, 3 and 6 weeks	51	Observational study
26	Kinney JS (15)	2011	USA	Periodontology	Periopathogens, Osteoprotegerin (OPG), MMP-9, MMP-8, IL-1β, Calprotectin, ICTP	Bi-monthly over 12 months	100	Observational study
27	Kochurova EV (44)	2017	Russia	Prosthodontics	MMP-2,MMP-8, MMP-9, TIMP-1, TIMP-2	Baseline, 2 weeks, 3 months	89	Observational study
28	Koppolu *P* (45)	2021	Saudi Arabia and India	Periodontology	Alkaline Phosphatase, Acid Phosphatase	Baseline, 4 weeks	135	Observational study
29	Kuboniwa M (46)	2016	Japan	Periodontology	Metabolites assessed by GC-MS (best fitting: Cadaverine, 5-Oxoproline, Histidine)	Baseline, 15 min	19	Observational study
30	Lee CH (47)	2018	Taiwan	Periodontology	IL-1β, IL-1ra, IL-6, IL-8, Platelet-Derived Growth Factor-BB, Vascular Endothelial Growth Factor, MMP-8, MMP-9, C-Reactive Protein, Lactoferrin	Baseline, 15 min	53	Observational study
31	Liu KH (48)	2016	South Korea	Periodontology	MMP-8, MMP-9, IL-1β in GCF, Nicotine, Cotinine, Hydroxy-Cotinine in saliva	Baseline, 2, 4 and 6 weeks, 12 months	122	Observational study
32	Morelli T (49)	2014	USA	General Dentistry	IL-1β, IL-1ra, IL-8, MCP-1, MMP-1, MMP-3, MMP-8, MMP-9, TIMP-1 through TIMP- 4, Neutrophil Gelatinase-Associated Lipocalin (NGAL)	Baseline, 1, 2 and 3 weeks	168	Observational study
33	Nascimento GG (50)	2019	Denmark	Periodontology	Myeloperoxidase (MPO), Neutrophil Elastase (NE), Soluble Urokinase-type Plasminogen Activator Receptor (suPAR), MMP-8, TIMP-1	Baseline, 1, 2 and 3 weeks	42	Observational study
34	Nishida N (51)	2008	Japan	Periodontology	Periopathogens, AST, Lactoferrin, Prostaglandin E2, IL-1β, MMP-8, MMP-9, IgA, Albumin	Baseline, 1 and 2 years	273	Observational study
35	Novakovic N (52)	2014	Serbia	Periodontology	Total antioxidant capacity, Albumin, Uric Acid, Superoxide Dismutase, Glutathione Peroxidase	Baseline, 2 months	63	Observational study
36	Oktay S (53)	2020	Turkey	Periodontology	Sialic Acid	Baseline, 3 months	40	Observational study
37	Önder C (54)	2017	Turkey	Periodontology	Malondialdehyde (MDA), 8-Hydroxy-Deoxy-Guanosine (8-OHdG), 4-Hydroxy-2-Nonenal (4-HNE)	Baseline, 6 weeks	51	Observational study
38	Öngöz Dede F (55)	2016	Turkey	Periodontology	8-Hydroxy-Deoxy-Guanosine (8-OHdG)	Baseline, 4 weeks	90	Observational study
39	Park JY (56)	2021	Korea	Periodontology	IL-1β, IL-6, MMP-8, MMP-9	Baseline, 4 and 8 weeks	104	Observational study
40	Parawani SR (57)	2012	India	Periodontology	Nitric Oxide (NO)	Baseline, 3 and 6 weeks	90	Observational study
41	Prakasam S (58)	2014	India	Periodontology	IL-4, IL-6, IL-10, IL-17, sTLR-2, sCD14	Baseline, 1 and 6 weeks	40	Observational study
42	Raghav D (59)	2017	India	Prosthodontics	IL-1β, Osteoprotegerin (OPG), MMP, Periopathogens	Baseline, up to 1 year	60	Observational study
43	Rabelo MS (60)	2021	Brazil	Periodontology	IL-1β, IL-8, IL-6, IL-2, IL-5, IL-4, IL-10, Interferon Gamma (IFN-*γ*), Granulocyte Macrophage Colony-Stimulating Factor (GM-CSF), TNF-α	Baseline, 30 days	60	Observational study
44	Ramseier CA (61)	2021	Switzerland	Periodontology	MMP-3, MMP-8, IL-1β	Baseline, 2,4 and 8 weeks	60	RTC
45	Rangbulla V (62)	2017	India	Periodontology	IgA, IL-1β, MMP-8	Baseline, 12 weeks	50	Observational study
46	Saloom HF (63)	2017	London	Orthodontics	Adipokines Leptin, Resistin, Myeloperoxidase (MPO), Cytokine Receptor for Nuclear Factor Kappa-B Ligand (RANKL)	Baseline, 1 h, 1 week	55	Observational study
47	Sanchez GA (64)	2013	Argentina	Periodontology	IL-1β, Prostaglandin E2	Baseline, 3 months	74	Observational study
48	Sexton WM (65)	2011	USA	General Dentistry	IL-1β, IL-8, Macrophage Inflammatory Protein (MIP) 1α, MMP-8, Osteoprotegerin (OPG), TNF-α	Baseline, 16 and 28 weeks	33	Observational study
49	Silbereisen A (66)	2020	Sweden	Periodontology	Molecular MMP-8, TIMP-1	Baseline, 7, 14, 21 and 35 days	10	Observational study
50	Syndergaard B (67)	2014	USA	Periodontology	IL-1β, IL-6, MMP-8, Macrophage Inflammatory Protein (MIP) 1α, Prostaglandin E2	Baseline, 7, 10 and 30 days	80	Observational study
51	Tatarakis N (68)	2014	USA	Periodontology	Osteoprotegerin (OPG), IL-4, IL-10	Baseline, 1 year	32	Observational study
52	Varghese J (69)	2020	India	Periodontology	8-Hydroxy-Deoxy-Guanosine (8-OHdG)	Baseline, 3 months,	40	Case-Control study
53	Venza M (70)	2006	Italy	Periodontology	Histamine	Baseline, 6, 12 and 24 months	125	Observational study
54	Wu J (71)	2018	China	Orthodontics	Different peptide profiles assessed by Maldi-Tof	Baseline, 1, 2 weeks, 1, 2, 6 months	36	Observational study
55	Yarkac F (72)	2018	Turkey	Periodontology	IL-1β, IL-10, salivary Chromogranin A (CgA)	Baseline, after periodontal therapy	60	RCT
56	Yoshida RA (73)	2019	Brazil	Periodontology	IL-1β, IL-6, IL-8, IL-10, TNF-α, IL-12p70	Baseline, 15 days	38	Observational study
57	Yoshie H (74)	2007	Japan	Periodontology	Aspartate Aminotransferase, Alanine Aminotransferase, Lactate Dehydrogenase	Baseline, 4 weeks	49	Observational study

## Results

### Included studies

**Figure 1:**
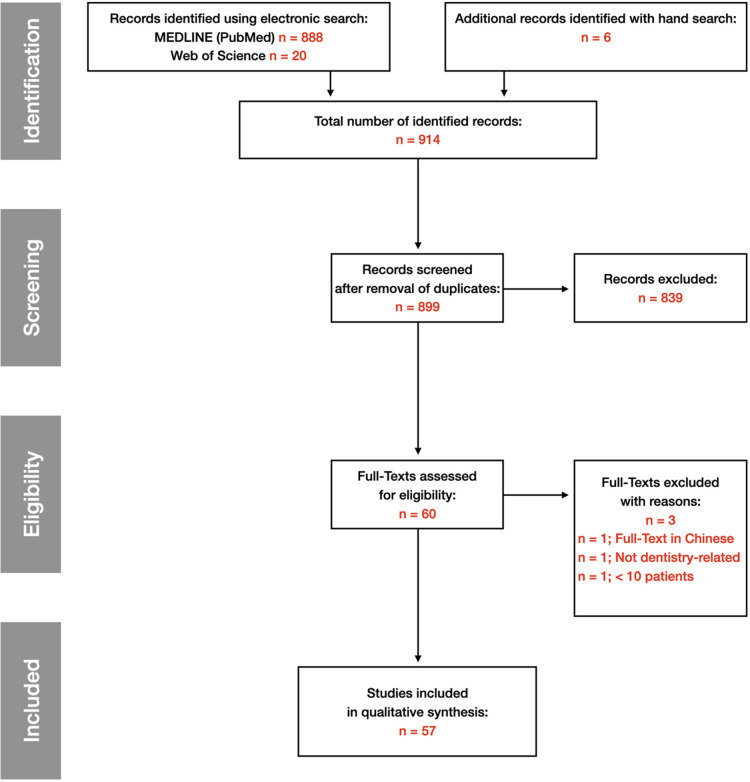
Search flowchart from identification to included studies.

The Scoping Research was completed on 2022-06-30 and results are current as of this date. Of the 914 titles retrieved by the search, 899 abstracts were further screened, and successively, 60 full-texts identified. A total of 3 full-texts were excluded from the final analysis. Finally, 57 full-texts were included for data extraction (Figure 1).

Included studies were judged to be of sufficient quality considering the specific study design. Detailed information of each study is tabularized for general data in Table 1 (author, year of publication, country of origin, then categorized in study design, dental disciplines including topics investigated and target biomarkers, monitoring intervals, total number of patients, and outcome metrics).

The publication dates range from 2000 to 2022 with continuously increasing numbers in recent years. Study types were categorized in observational clinical studies (*n* = 50), RCTs (*n* = 4), case-control studies (*n* = 2), and pilot study (*n* = 1). A total of 4,125 patients were investigated with patient monitoring intervals from 2 h up to 3 years, depending on the respective trial design and focused research question. Dental disciplines involved were periodontology (*n* = 42), general dentistry (*n* = 7), orthodontics (*n* = 3), prosthodontics (*n* = 2), oral surgery (*n* = 1), implantology (*n* = 1), and temporo-mandibular disorders (*n* = 1). As explanation, the term “general dentistry” has been used for diagnostic or therapy protocols fundamental to protecting and maintaining a good standard of oral health, but not related to any dental specialty.

**Figure 2:**
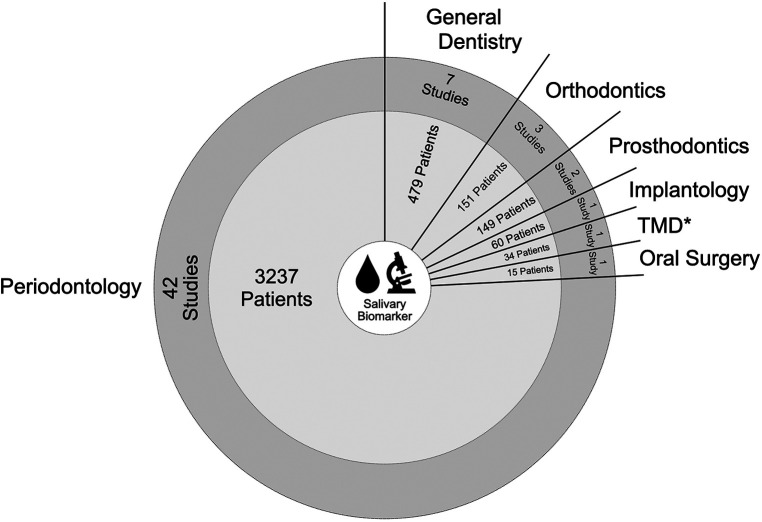
Map of data items associated with number of studies related to dental disciplines including patients involved (* TMD = temporo-mandibular disorder).

**Figure 3:**
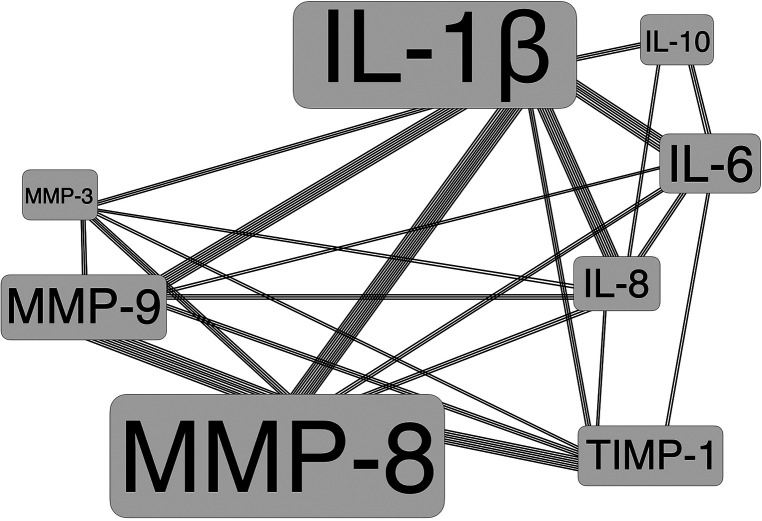
Major salivary biomarkers with frequency of occurrence (as illustrated by font size) and their combinations among studies.

Depending on these classified dental disciplines, a variety of topics with diverse salivary biomarkers using different research techniques and monitoring intervals were reported. Here, main focus of salivary biomarkers investigated was on Matrix-Metalloproteinases (MMP) plus Interleukins (IL). Figure 2 shows a map of disciplines by number of studies and patients included in this Scoping Review, and Figure 3 displays the major salivary biomarkers and their distribution within the studies.

Due to the pronounced heterogeneity of the included studies, a direct comparison among the identified publications was not deemed possible. Therefore, this Scoping Review of the included full-texts followed a descriptive analysis. Supplementary_Data 3 summarizes the detailed information of the included studies.

## Discussion

The aims of this Scoping Review were: (i) to compile studies on salivary biomarkers in terms of personalized oral healthcare and precision dentistry, (ii) to investigate the clinical relevance for oral health, and (iii) to summarize an outlook for future developments based on these results. “Scoping Reviews are also executed in a systematic, replicable manner, but usually intended to identify the types of available evidence in a given field and to discover knowledge gaps, to clarify concepts and definitions, and to examine how research is conducted, eventually, inform research, educational and clinical policy and priorities” (75, 76). A Scoping Review aims to “map the literature on a particular topic or research area and provide an opportunity to identify key concepts, gaps in the research; and types and sources of evidence to inform practice, policymaking, and research” (77). Therefore, the specific format Scoping Review was chosen to screen this relatively young topic and to summarize the current state of research in the context of a broad overview. Although a preliminary review protocol is available as Supplementary_Data 2, it should be noted that this Scoping Review was not registered on an online platform.

In general, precision dentistry provides diagnostic or therapeutic protocols that are as individualized as the disease with specific signs and symptoms. This approach is based on the identification of clinical information enabling the understanding of the patients' unique genomic constitution and how that makes them vulnerable to certain diseases. Each patient is to be treated with comprehensive consideration of individual circumstances using multidisciplinary channels, beyond the solely functional aspect of disease diagnosis. This also includes the continuous adjustment of therapy to keep pace with the progress of dental and medical knowledge (78, 79). It is still a very recent development: the move from purely evidence-based to personalized dentistry – and the meaning (and importance) for the dental sub-disciplines is entirely different.

Several research groups all over the world concentrate on personalized oral healthcare and precision dentistry today. The topic is of great interest in the field of dentistry, although there is (still) an imbalance in the distribution of the dental disciplines involved. The majority of all included publications were assigned to the field of periodontology, representing a proportion of 74% related to the number of studies, or 79% related to the number of patients, respectively. In restorative and reconstructive dentistry, research on salivary biomarkers seems to play a minor role at the present time. A possible explanation could be that periodontal diagnostics and treatment is very standardized following generally accepted principles (80). Therefore, it is much easier to implement personalized workflows in periodontology compared to other disciplines. For example, in reconstructive dentistry, it is *per se* a highly personalized discipline and uniform standard operating procedures (SOP) are hard to implement, except for situations that are directly comparable, such as complete edentulous patients (81).

The main causes of tooth loss are caries and periodontitis (82). Caries can usually be prevented very well by the patients' self-discipline with oral hygiene devices and the use of fluoride-containing toothpaste combined with mouth rinses. For periodontitis, however, clinical study outcomes could help to understand closer associations of genetic factors in periodontitis patients (83). It is therefore not surprising that with the further development of laboratory methods in recent years, salivary biomarkers have increasingly become the focus of periodontal research (84).

### What saliva biomarkers have been investigated in clinical trials in terms of personalized oral healthcare and precision dentistry mapped for disciplines and/or indications?

The abundance of matrix metalloproteinases (MMP) and interleukins (IL) as salivary biomarkers was particularly striking in this Scoping Review. It was also observed that a variety of different combinations of MMP and IL were examined. Most commonly, IL-1β, MMP-8, IL-6, and MMP-9 were determined in saliva, followed by IL-8, TIMP-1, IL-10 and MMP-3. Altogether, more than 92 different biomarkers were analyzed in saliva. This leads to the conclusion that currently no consensus exists on which biomarkers should be used for what specific scientific target(s) and with which intention.

Therefore, no clear recommendations can be given related to specific salivary biomarkers associated for personalized oral healthcare principles at this time. MMP and IL seem to be the most promising biomarkers, in particular in periodontology.

### What has been the clinical relevance for oral health and its correlation to systemic health?

An exciting field also seems to be the pre-therapeutic examination of saliva for risk assessment of patients with regard to specific periodontal treatment modalities. This represents a true evolution from purely evidence-based (always applied in the same way) approaches to personalized treatment cascades. It remains very exciting whether new treatment concepts can be derived from this in the future, such as the creation of pre-therapeutic risk profiles of individual patients. Training datasets could be used to compute predictive biomarkers using statistic model predictions and clinical assessments to either differentiate health conditions or to predict treatment outcomes (85). Ideally, clinical measurements, applied threshold values, handling of missing data or data below the detection limit should be described thoroughly and very precisely to allow generalizability.

Only few studies in this Scoping Review examined a possible association of oral and systemic health (*n* = 6): most common was obesity/nutrition (19, 55, 56), followed by diabetes mellitus (59, 68, 70), and influence of pregnancy (72).

### Outlook to the future

Although salivary biomarker research appears to be extremely promising, it remains to be seen to what extent the MedTech industry will jump on this technology in the future (86). Without an economic driver, it will be difficult to further investigate this costly research topic. Besides periodontology, peri-implantitis might also be an exciting field. This is certainly also in the interest of implant manufacturers, so that financial support for research would be guaranteed. Nevertheless, the results of this Scoping Review revealed only 1 clinical trial focusing on saliva and possible association with peri-implantitis (21). In addition, dentistry could become the door-opener for routine diagnostics in medicine, e.g., assessment of glucose concentration in diabetes patients (84).

Unfortunately, the studies identified demonstrated heterogeneous quality standards, starting with the study design, the number patients included, the salivary biomarkers investigated, and monitoring intervals. Direct comparisons are not possible. It would certainly be helpful if biomarkers could be defined (according to the classification in the different dental disciplines) and then examined under standardized conditions in various clinical studies.

Salivary biomarkers have the potential to change the field of dentistry in all disciplines. The increasing interest in dental research is obvious, demonstrated by the growing number of publications in recent years. At this time, however, the predominant discipline is periodontology. Precision dentistry and personalized workflows are trendy buzzwords, the future research will proof, if the high expectations can be fulfilled. Several research groups investigating diverse salivary biomarkers in a variety of combinations. The limiting factor of Big Data research is the amount of structured data available (87, 88). Therefore, the establishment of an open research data community comprising information of salivary samples could help to foster the further development of personalized oral healthcare and precision dentistry.

## Data Availability

The original contributions presented in the study are included in the article/**Supplementary Material**, further inquiries can be directed to the corresponding author/s.
