# Supplementary material for: Salivary biomarkers as key to monitor personalized oral healthcare and precision dentistry: A scoping review
Source: Front Oral Health. 2022 Sep 22;3:1003679. doi: 10.3389/froh.2022.1003679 (PMC9632857; doi:10.3389/froh.2022.1003679)
Supplement: Supplementary file 1 [file Table1.docx]

**Systematic search strategy – Scoping Review (PCC)**

| **Authors** | Pune Nina PAQUE, Jenni HJERPPE, Anina ZUERCHER, Ronald JUNG, Tim JODA | |
| --- | --- | --- |
| **Focused question** | **Are saliva (P) biomarkers (C) the key to personalized oral healthcare and precision dentistry(C)?**  (What are the salivary biomarkers for oral and systemic diseases?)  *A three-pronged search strategy was applied, combining the source of analysis [“Saliva”], the technique of interest [“Biomarkers”], and the field of interest [“(Personalized) Dentistry” / “Precision Dentistry”].* | |
| **Search Strategy** | **Source of Analysis** (=**P**opulation) | #1 – “Saliva” |
|  | **Concept** | #2 – “Biomarker” |
|  | **Context** | #3 – “(Personalized) Dentistry” OR “Precision Dentistry” |
|  | **Search combination** | **#1 AND #2 AND #3**  Search: (saliva) AND (biomarker) AND ((personalized dentistry) OR (precision dentistry))  ("saliva"[MeSH Terms] OR "saliva"[All Fields] OR "salivas"[All Fields] OR "saliva s"[All Fields] OR "salivary"[All Fields]) AND ("biomarker s"[All Fields] OR "biomarkers"[MeSH Terms] OR "biomarkers"[All Fields] OR "biomarker"[All Fields]) AND ((("person s"[All Fields] OR "personable"[All Fields] OR "personableness"[All Fields] OR "personal"[All Fields] OR "personalisation"[All Fields] OR "personalise"[All Fields] OR "personalised"[All Fields] OR "personalising"[All Fields] OR "personality"[MeSH Terms] OR "personality"[All Fields] OR "personalities"[All Fields] OR "personality s"[All Fields] OR "personalization"[All Fields] OR "personalize"[All Fields] OR "personalized"[All Fields] OR "personalizes"[All Fields] OR "personalizing"[All Fields] OR "personally"[All Fields] OR "personals"[All Fields] OR "persons"[MeSH Terms] OR "persons"[All Fields] OR "person"[All Fields]) AND ("dentistry"[MeSH Terms] OR "dentistry"[All Fields] OR "dentistry s"[All Fields])) OR (("precise"[All Fields] OR "precised"[All Fields] OR "precisely"[All Fields] OR "preciseness"[All Fields] OR "precises"[All Fields] OR "precision"[All Fields] OR "precisions"[All Fields]) AND ("dentistry"[MeSH Terms] OR "dentistry"[All Fields] OR "dentistry s"[All Fields]))). All publications were included until the 30th of June 2022. The search in Web of Science was conducted with the terms “(ALL=(saliva*biomarker* ((person* and dentistry*) or (precis* and dentistry*)))). |
| **Database search** | **Electronic** | MEDLINE via PubMed Web of Science |
|  | **Hand search (Journals)** | t.b.d. |
| **Selection criteria** | **Inclusion criteria** | Clinical Trials (at least 10 patients) |
|  | **Exclusion criteria** | In-Vitro, Laboratory, and Animal Studies |
